# Limited Mobility to the Bed Reduces the Chances of Discharge and Increases the Chances of Death in the ICU

**DOI:** 10.3390/clinpract12010002

**Published:** 2021-12-21

**Authors:** Talita Leite dos Santos Moraes, Joana Monteiro Fraga de Farias, Brunielly Santana Rezende, Fernanda Oliveira de Carvalho, Michael Silveira Santiago, Erick Sobral Porto, Felipe Meireles Doria, Kleberton César Siqueira Santana, Marcel Vieira Gomes, Victor Siqueira Leite, Reuthemann Esequias Teixeira Tenório Albuquerque Madruga, Leonardo Yung dos Santos Maciel, Juliana Dantas Andrade, Jader Pereira de Farias Neto, Felipe J. Aidar, Walderi Monteiro da Silva Junior

**Affiliations:** 1 Graduate Program in Physical Education, Federal University of Sergipe (PPGEF-UFS), São Cristovão 49100-000, SE, Brazil; joanamfraga@hotmail.com (J.M.F.d.F.); brunyrezende@hotmail.com (B.S.R.); michaelquadril1308@gmail.com (M.S.S.); fjaidar@gmail.com (F.J.A.); walderim@yahoo.com.br (W.M.d.S.J.); 2 University Hospital, Federal University of Sergipe (HU/UFS), Aracaju 49060-108, SE, Brazil; fsoliveira.fisio@gmail.com (F.O.d.C.); judantasandrade@gmail.com (J.D.A.); 3 Department of Medicine, Tiradentes University (UNIT), Aracaju 49032-490, SE, Brazil; ekporto@hotmail.com; 4 Group of Studies and Research of Performance, Sport, Health and Paralympic Sports (GEPEPS), Federal University of Sergipe (UFS), São Cristovão 49100-000, SE, Brazil; felipemdoria@gmail.com; 5 Military Police Hospital, Aracaju 49072470, SE, Brazil; k.cesar@uol.com.br; 6 Program of Traumatology and Orthopaedics, Federal University of Sergipe (UFS), São Cristovão 49100-000, SE, Brazil; vieiramarcelgomes@gmail.com (M.V.G.); victor_s_leite@hotmail.com (V.S.L.); 7 Department of Medicine, Federal University of Sergipe (UFS), São Cristovão 49100-000, SE, Brazil; fattam@uol.com.br; 8 Department of Physiotherapy of Lagarto, Federal University of Sergipe (UFS), Lagarto 49400-000, SE, Brazil; yung_maciel@hotmail.com; 9 Department of Physiotherapy, Federal University of Sergipe (UFS), São Cristovão 49100-000, SE, Brazil; jadernetofisio@hotmail.com

**Keywords:** mobility limitation, intensive care unit, early ambulation

## Abstract

Background: Progressive mobility in the ICU has been recommended; however, the definitions of low, moderate, and high mobility in the ICU still diverge between studies. Therefore, our objective was to classify the mobility of the sample from verticalization and active withdrawal from the bed, and from that, to analyze the chances of discharge, death, and readmission to the ICU. Materials and methods: This is an observational and retrospective study that consults the medical records of individuals admitted to the ICU of the University Hospital of Sergipe (HU/SE) between August 2017 and August 2018. Mobility level was classified based on the Intensive Care Unit Mobility Scale (IMS). Results: A total of 121 individuals were included. The mean age was 61.45 ± 16.45, being 53.7% female. Of these, 28 (23.1%) had low mobility, 33 (27.3%) had moderate mobility, and 60 (49.6%) had high mobility. Individuals with low mobility were 45 times more likely to die (OR = 45.3; 95% CI = 3.23–636.3) and 88 times less likely to be discharged from the ICU (OR = 0.22; 95% CI = 0.002–0.30). Conclusion: Those who evolved with low mobility had a higher chance of death and a lower chance of discharge from the ICU. Moderate and high mobility were not associated with the investigated outcomes.

## 1. Introduction

The assessment of mobility of individuals hospitalized in intensive care units (ICUs) has been recommended to quantify the responses to therapies, evolution, or functional decline of individuals [1]. The independence to move and transfer postures should be part of the goals outlined by the multidisciplinary team, as well as the reduction of length of stay, especially in mechanical invasive mechanisms, lower rates of death, and readmission, all of which influence the reduction of costs related to hospitalization in the intensive care unit (ICU) and hospital in general [1,2].

Even in an institution where there are early mobilization protocols and in the face of recommendations for the insertion of mobilization protocols at systematic levels of progression of postures and activities, studies have shown that the highest level of mobilization achieved by most individuals, prior to discharge from the ICU, has been turning over in bed, corresponding to 57.5% of the sample [1,3,4]. Furthermore, the incidence of activities outside the bed has been low (25%), with only 5% of individuals performing some activity away from the bed in the ICU [3].

Early mobilization in the ICU is not always synonymous with high mobility in these units [4,5]. Although they may be associated, the individual can be mobilized within the first 48 or 72 h after admission to the ICU, but not performing orthostasis or walking, by various factors [4,5,6,7]. It has been suggested that the multidisciplinary team work with progressive mobility goals, culminating in out-of-bed mobilization, so that in addition to reducing the length of stay in the ICU, they favor greater mobility, aiming to improve individual-centered outcomes such as improved function and quality of life post discharge [4,5,8].

There is a conflict between the articles regarding the cutoff point in the IMS scores to classify the mobility levels of those in the ICU with low or high mobility, for example. There was a clinically significant difference; according to the study by Claire (2018), it ranges from 1.4 to 3 points on the Intensive Care Unit Mobility Scale (IMS), which is one of the main mobility assessment scales in the ICU. It has been used as a binary variable, defined as passive mobilization or active mobilization, not making it possible to analyze whether the level of care and the performance of activities in and out of bed influence the results in the ICU [9,10,11]. That said, the aim of the present study was to classify the level of mobility of the sample based on verticalization and active distancing from the bed through the IMS score and from this analyze the chances of discharge, death, and readmission of individuals admitted to the ICU.

## 2. Materials and Methods

### 2.1. Study Design

This is an observational and retrospective study, carried out from the consultation of records in the medical records of hospitalized individuals, from August 2017 to August 2018. The recommendations of the STROBE Statement for cross-sectional studies were followed [12].

The present study was approved on 17 September 2018 by the Ethics and Human Research Committee of the Federal University of Sergipe (UFS) with the technical advice number 2,897,651.

### 2.2. Search Location

Data collection was carried out in the ICU of the University Hospital of Sergipe (HU/SE), which has the characteristic of a mixed ICU and serves individuals with an elective clinical and surgical profile, with a predominance of abdominal surgeries.

The aforementioned ICU had five beds, and it also had a culture of progressive mobility already established through an institutional protocol, in which, after finding the individual fit for increased mobility, multidisciplinary strategies were drawn up for the performance of bedside sitting, progressing to orthostasis and stationary gait and subsequently assisted or independent walking for at least five meters.

### 2.3. Participants

Initially, the medical records of all individuals aged ≥ 18 years were selected, both sexes, admitted to the ICU for medical or elective surgical reasons, under use or not of invasive mechanical ventilation. Those with a diagnosis of neurodegenerative disease were not included, be it pre or after admission to the ICU, in the postoperative period of spine and/or lower limb fractures, amputation of one or both lower limbs, diagnosis of cerebrovascular accident in the acute or chronic phase or in which any conditions that contraindicate or make it impossible to increase mobility were presented, hospitalization time ≤ 24 h. Medical records with missing data were excluded.

### 2.4. Study Sample

**Figure 1:**
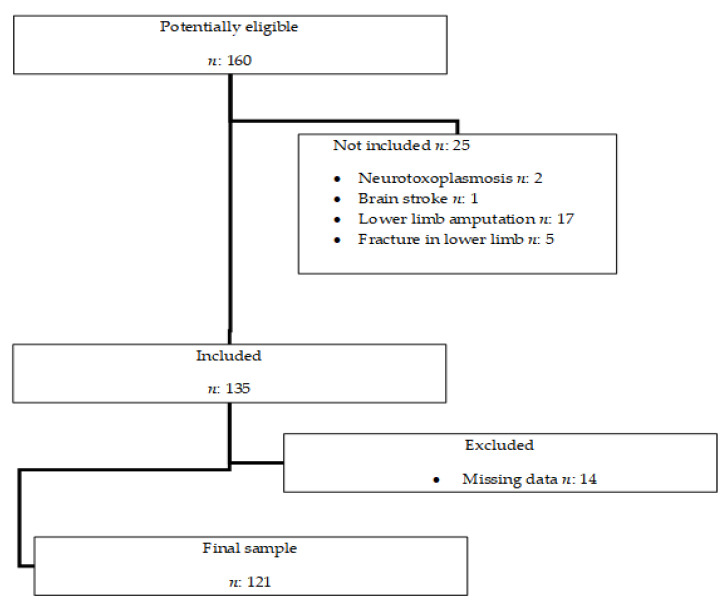
Study sample selection flowchart.

The non-probabilistic sample, for convenience, was initially made up of all individuals who were admitted to the ICU in the period previously established for the study, of which the medical records were available for data collection, totaling 160 medical records. Figure 1.

### 2.5. Instruments and Procedures

The medical records in the hospital’s archive sector were checked and data from an assistance instrument entitled Physical Therapy Indicators in the ICU were selected, which is used for the assessment and monitoring of individuals in that ICU.

Data were collected: age, gender, reason for admission, admission profile (clinical or surgical), mobility (IMS score), use of invasive ventilatory support (pre- or post-admission), length of stay, time on invasive mechanical ventilation (IMV), early mobilization and outcomes (readmission, discharge from the ICU and death). The records of the IMS score were performed by the team’s physical therapists in all shifts until discharge from the unit, death, or transfer of the patient.

Classification of mobility level considered the highest IMS score achieved by the individual during the entire ICU stay. An IMS score of 0–3 was considered low mobility, 4–6 was considered moderate mobility and 7–10 was considered high mobility based on the patient’s verticalization and distance from the bed. For individuals who had the greatest mobility to sit on the bedside (partial verticalization), low mobility was considered. Those who underwent orthostasis at the bedside, had stationary gear, or were passively transferred to the seat (they performed the complete verticalization but remained close to the bed) were classified as having moderate mobility. Those who walked, even if assisted, for more than 5 m in the unit (complete verticalization and active/assisted distancing from the bed) were classified as high mobility. A 3-point difference in the IMS between low and moderate mobility levels was also considered, as well as between moderate mobility and high mobility, considering the clinically relevant difference in points between the levels [10].

### 2.6. Studied Variables

The level of mobility was considered a predictor variable. Gender, identity, admission profile (clinical or surgical), length of stay, time in IMV, and early mobilization were considered possible influencers on the level of mobility. The outcome data were discharge from the ICU, readmission, and death.

### 2.7. Statistical Analysis

The IMS score was expressed as median and percentiles. Continuous variables are presented as mean ± standard deviation. Categorical variables were expressed in absolute and relative frequencies.

Due to the dichotomous characteristics of the outcome variables, all analyses were performed using non-parametric tests, and it is not necessary to analyze the normality of the sample. Possible differences in mobility level between admission, clinical, and surgical profiles were verified using the Wilcoxon test. Possible influences of gender variables, age, admission profile (clinical or surgical), length of stay, time on mechanical ventilation, and early mobilization in mobility levels and their association with outcomes were verified from the crude and adjusted association analysis. *p* < 0.20 was considered for inclusion of the variable in the adjusted model. The estimation of odds and probability of outcomes were performed using simple logistic regression. The results of the analyses were considered significant when *p* < 0.05, and the entire analysis was performed using the SPSS software, version 22.0 (IBM SPSS Statistics for Windows IBM Corp, Released 2013, Armonk, NY, USA).

## 3. Results

Initially, 160 potentially eligible individuals were identified. After detailed analysis of the records, data from 121 of them were included in the study.

In Table 1, the descriptive characteristics of the sample are included. It was found that 86 individuals, 71.08% of the sample, had a surgical profile. Individuals with a clinical profile had longer stays in the ICU and longer on MV.

Individuals with a surgical profile had a significantly higher mobility level compared to clinicians (z = −9.72; *p* < 0.001), with a median of IMS 4 (p25: 3; p75: 8) for clinicians and 8 (p25: 6; p75: 8) for the surgical ones. In the overall sample, 28 individuals (23.1%) had low mobility, 33 individuals had moderate mobility (27.3%), and 60 had high mobility (49.6%).

**Figure 2:**
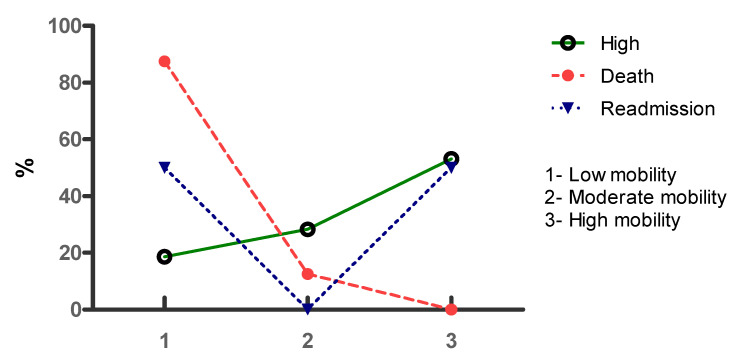
Discharge, death, and readmission according to the mobility level of individuals admitted to an ICU.

Of the individuals who were discharged from the ICU, 21 had low mobility (18.6%), 32 had moderate mobility (28.3%), and 60 had high mobility (53.1%). Among those who had death as an outcome, seven had low mobility during their ICU stay (87.5%), only one had moderate mobility (12.5%), and there was no high mobility in the ICU in this group. Of the individuals who were readmitted, three had high mobility (50%) and three had low mobility (50%). The percentages of discharge, death, and readmission by mobility level are shown in Figure 2.

In the group of individuals who required the use of IMV, 11 evolved with low mobility (44%), nine with moderate mobility (36.6%), and five (20%) with high mobility. The level of mobility was associated with the outcomes of discharge and death in the sample in general, being x^2^(df) = 2; *p* < 0.01; r = 0.40 x^2^(df) = 2; *p* < 0.01; r = 0.40 respectively. Regarding the rehospitalization outcome, there was no significant association with x^2^(df) = 2; *p* < 0.10; r = 0.19.

Younger individuals were less likely to develop low mobility (OR: 0.95; 95% CI = 0.92–0.98). Early mobilization generated 64 less chances (OR: 0.36; 95% CI = 1.05–12.95) and the surgical profile generated 81 less chances of low mobility level (OR: 0.19; 95% CI = 0.06–0.62) (Table 2).

Table 3 presents a crude and adjusted analysis of the level of mobility with the outcomes of discharge, death, and readmission to the ICU. The analysis showed that individuals with a low level of mobility are almost 45 times more likely to progress to death (OR = 45.3; 95% CI = 3.23–636.3) and 88 times less likely to progress to discharge from the hospital. ICU (OR = 0.22; 95% CI = 0.002–0.30), both with *p* < 0.05. After adjustment, moderate and high mobility levels were not associated with the investigated outcomes.

## 4. Discussion

The main findings of the present study were that individuals with low mobility were 88 times less likely to be discharged from the ICU and 45 times more likely to evolve to death in the ICU. This research assessed the level of mobility of individuals using a specific instrument and establishing cutoff points for their classification based on the individual’s verticalization and ability to actively move away from bed, and considering the clinically significant difference of 3 points between the highest IMS score in each level [10].

Previous studies such as those by Luque et al. and Frazzitta et al. reported the importance of early complete verticalization in the ICU, even in a passive way [13,14]. Prolonged time in the sitting or reclining posture during waking hours is related to mortality in the general population; however, passive verticalization does not seem to generate effects superior to conventional protocols that include mobilization in bed [14,15].

Growing evidence indicates that early mobilization practices minimize impairments in the mobility of individuals in ICUs [14,15,16]. Therefore, early mobilization protocols are implemented as part of the routine of these units as a strategy to increase the mobility of individuals, which starts with passive movement in bed in bed, but it requires progression to antigravity postures, with systematically increased levels of mobilization, which culminate in the removal of the patient from the bed [1,17,18].

Studies show that activities such as assisted or independent walking in the ICUs are still related to younger individuals, which corroborates our findings, in which younger individuals are less likely to evolve with low mobility [1,17]. Although it is well established in the literature that immobility contributes to an increased risk of delirium, aspiration pneumonia, pressure ulcers, muscle weakness, and prolonged hospital stay, among other factors, most elderly people are encouraged to stay in bed, with the main factor the prevention of falls during hospitalization, even the elderly are at increased risk of developing sarcopenia, which is an important marker of functional decline [4,19,20,21].

In the practice of progressively increasing mobility, the evolution from the decubitus position to bedside sitting is one of the first activities performed from the moment they acquire clinical and hemodynamic stability, characterizing the initial kick-off of increased mobility in the ICU [4,10]. Evolving an individual from the decubitus position to bedside sitting reduces the time at rest, favors pulmonary ventilation, stimulates cervical and trunk control, contributes to neurological and cardiovascular adaptations, and allows the individual to perform limb and trunk movements in a more functional form than in decubitus [20]. However, the results of the present study suggest that it is not enough to favor greater chances of hospital discharge, although there are conditions intrinsic to the individuals, such as the presence of accesses, tubes, and chest tubes, which can be interpreted as barriers to increased mobility [15,22,23].

Similarly, activities that involve complete verticalization while still close to the bed, such as orthostasis, transfer from bed to chair, and stationary gait, considered in this study as moderate mobility, are performed as a form of mobility progression until the individual becomes able to walk away assisted or actively under bed supervision [20,24]. Although with greater benefits than low mobility, the percentages of death and readmission were still higher than in the high mobility group, who walked in the unit, with walking perhaps the key to minimizing the chances of negative outcomes in the ICU [20,24].

Jesus et al. justified in their study that the 14.3% decline in mobility in their sample of individuals admitted to the ICU was due to the predominance of surgical individuals. However, in the results of the present study, individuals with a surgical profile had a higher median mobility. Most elective surgical individuals are subjected to the use of IMV, mostly, just to enable deep sedation and anesthesia to perform the surgical procedure [25,26]. This fact favors that after stabilizing the condition, the individual progresses to simple weaning from IMV, increased mobility, and improvement in the level of physical activity in the ICU at an early stage, which corroborates the statement by Stamatakis et al. that individuals with less severe organic insufficiencies evolve with greater mobility [3,27,28].

This research has some limitations. The smaller number of clinical individuals compared to surgical individuals and the small number of beds in the ICU where the study took place may have favored a low percentage of death and readmission to the selected ICU, limiting the generalization of some results.

In the present study, it was not possible to estimate the chances of death in individuals who evolved with high mobility, since deaths in this group were non-existent. Then, it is questioned whether the high level of mobility has a causal effect on the absence of deaths found, which suggests the carrying out of future studies with similar objectives, with percentages of more balanced outcomes, in order to confront such results.

Considering that one of the exclusion criteria was individuals who had some condition that interfered with the measurement of the level of mobility, and considering that the IMS has high agreement with movement sensors, future studies can be carried out using these sensors in order to quantify the mobility of individuals with a previous or acquired change in the ICU, which cannot be measured using the IMS.

## 5. Conclusions

Individuals who had low mobility, who tended to sit at the bedside, assisted or active, but who did not undergo orthostasis during the ICU stay had 45 times more chance of evolving to death and 88 times less chance of being discharged from the hospital. ICU younger individuals, those who underwent early mobilization, and those who were hospitalized with a surgical profile, were less likely to progress to low mobility.

In individuals who evolved with a moderate level of mobility, who underwent complete verticalization through orthostasis, stationary gait, and sitting in an armchair close to the bed, had lower percentages of death and readmission in comparison with low mobility. In the group with high mobility, deaths were non-existent. Both moderate mobility and high mobility were not associated with the investigated outcomes. A possible limitation of our study is the fact that the profile of the patients included in this sample had undergone the same surgical procedure.

## Figures and Tables

**Table 1:** Demographic and clinical characteristics of the studied population.

Variables	
Age (mean ± SD)	61.45 ± 16.45
Male (*n* (%))	56 (46.3)
Female (*n* (%))	65 (53.7)
Diagnostics *n* (%)	
IRpA	14 (11.57)
CPA	2 (1.66)
Angina pectoris	1 (0.82)
Sepsis	10 (8.26)
Pneumonia	2 (1.66)
Chronic lung disease	3 (2.48)
HDA/Duodenal ulcer	2 (1.66)
Miliary tuberculosis	1 (0.82)
Postoperative of abdominal surgeries	65 (53.72)
Postoperative of pelvic surgeries	9 (7.44)
Postoperative of pulmonary surgeries	6 (4.96)
Postoperative head and neck surgery	6 (4.96)
Length of stay (days)	
Clinical profile	9.25 ± 9.75
Curgical profile	2.70 ± 1.33
VMI time (days)	
Clinical profile	16.51 ± 18.98
Surgical profile	1.92 ± 1.50
ICU discharge *n* (%)	113 (93.4)
Readmission *n* (%)	6 (4.95)
Death *n* (%)	8 (6.6)

IRpA—acute respiratory failure; CPA—cardiorespiratory arrest; HDA—upper gastrointestinal bleeding; VMI—invasive mechanical ventilation; *n*—sample number; %—percentage; SD—standard deviation.

**Table 2:** Crude and adjusted association between factors and levels of mobility in the ICU.

Variable					Mobility Level							
	Low				Moderate				High			
	Raw		Adjusted		Raw		Adjusted		Raw		Adjusted	
	OR	95% CI	OR	95% CI	OR	95% CI	OR	95% CI	OR	95% CI	OR	95% CI
Sex												
Male	1											
Female	0.68	0.29–1.59			1.47	0.65–0.31			0.97	0.47–1.98		
Age												
Male	0.97	0.94–1.00 *	0.95	0.92–0.98	0.99	0.96–1.01			1.03	1.004–1.05	1.03	1.01–1.06
Female	0.99	0.93–1.07			0.92	0.85–0.99	0.92	08.5–1.007	1.12	1.00–1.23 *	1.00	0.90–1.10
Early mobilization												
No	1											
Yes	0.16	0.59–0.46	0.36	1.05–12.95	1.55	0.55–4.31			12.14	2.67–55.12	9.41	2.01–44.02
Profile												
Clinical	1											
Surgical	0.23	0.09–0.58	0.19	0.06–0.62	1.09	0.245–2.62			3.47	1.48–8.11	2.50	1.01–6.20
IMV time	0.15	0.96–1.23 *	0.95	0.88–1.04	1.00	0.95–1.06				0.86–1.01		

OR: odds ratio; CI: confidence interval; IMV: invasive mechanical ventilation; * *p* < 0.20.

**Table 3:** Crude and adjusted analysis of outcomes associated with exposure to low mobility, moderate mobility, and high mobility in the ICU.

					Mobility Level							
Outcomes	Low				Moderate				High			
	Raw		Adjusted		Raw		Adjusted		Raw		Adjusted	
	OR	95% CI	OR	95% CI	OR	95% CI	OR	95% CI	OR	95% CI	OR	95% CI
Discharge	0.03	0.004–0.27	0.22	0.002– 0.30	2.70	0.32– 23.38			1.15	1.04– 1.26	∞ ∞	
Death	30.66	3.57– 262.80	45.3	3.23– 636.38	0.36	0.43– 3.05			1.15	1.04– 1.26		

OR: odds ratio; CI: confidence interval; ∞—no maximum likelihood estimates, as all individuals progressed to discharge and none evolved to death. Outcomes adjusted for age, length of stay, early mobilization, and profile. The he “no” category was automatically fitted in the model as a reference.

## Data Availability

Data presented in this study are available upon request by the author for correspondence.
